# Vowel reduction in word-final position by early and late Spanish-English bilinguals

**DOI:** 10.1371/journal.pone.0175226

**Published:** 2017-04-06

**Authors:** Emily Byers, Mehmet Yavas

**Affiliations:** Linguistics Program, Florida International University, Miami, Florida, United States of America; University College London, UNITED KINGDOM

## Abstract

Vowel reduction is a prominent feature of American English, as well as other stress-timed languages. As a phonological process, vowel reduction neutralizes multiple vowel quality contrasts in unstressed syllables. For bilinguals whose native language is not characterized by large spectral and durational differences between tonic and atonic vowels, systematically reducing unstressed vowels to the central vowel space can be problematic. Failure to maintain this pattern of stressed-unstressed syllables in American English is one key element that contributes to a “foreign accent” in second language speakers. Reduced vowels, or “schwas,” have also been identified as particularly vulnerable to the co-articulatory effects of adjacent consonants. The current study examined the effects of adjacent sounds on the spectral and temporal qualities of schwa in word-final position. Three groups of English-speaking adults were tested: Miami-based monolingual English speakers, early Spanish-English bilinguals, and late Spanish-English bilinguals. Subjects performed a reading task to examine their schwa productions in fluent speech when schwas were preceded by consonants from various points of articulation. Results indicated that monolingual English and late Spanish-English bilingual groups produced targeted vowel qualities for schwa, whereas early Spanish-English bilinguals lacked homogeneity in their vowel productions. This extends prior claims that schwa is targetless for F2 position for native speakers to highly-proficient bilingual speakers. Though spectral qualities lacked homogeneity for early Spanish-English bilinguals, early bilinguals produced schwas with near native-like vowel duration. In contrast, late bilinguals produced schwas with significantly longer durations than English monolinguals or early Spanish-English bilinguals. Our results suggest that the temporal properties of a language are better integrated into second language phonologies than spectral qualities. Finally, we examined the role of nonstructural variables (e.g. linguistic history measures) in predicting native-like vowel duration. These factors included: Age of L2 learning, amount of L1 use, and self-reported bilingual dominance. Our results suggested that different sociolinguistic factors predicted native-like reduced vowel duration than predicted native-like vowel qualities across multiple phonetic environments.

## Introduction

Mastery of the stress pattern of American English is predicated on a speaker’s ability to assign stress to particular syllables using his/her linguistic knowledge of the phonological patterns of the language [1]. Stressed vowels are produced with greater intensity and duration than their unstressed counterparts, and are more perceptually salient to listeners [2,3]. In unstressed syllables, vowels are prevented from extending to the periphery of the oral cavity where most meaningful vowel contrasts occur. As a result, schwas are often regarded as the neutralization of vowel quality contrasts [2–4].

As unstressed vowels, schwas have been described as the product of a collapsed vowel space caused by reduced subglottal pressure, decreased muscular effort, and lack of coordinated gestures [3, 5–9]. These decreases in articulatory effort have the combined effect of localizing schwa in English’s underutilized central vowel space [2,5]. Where measurable, acoustic analyses generally corroborate phonological descriptions of schwa as the midpoint of the audible vowel space for F1(height) and F2 (backness) dimensions [10]. For example, prototypical English schwas spoken by an adult male are expected to contain formant bands at 500/1500/2500 Hz at the midpoint of periodicity [5]. While it is possible for peripheral vowels to undergo truncation or spectral-quality adjustment due to phonetic factors such as faster speaking rate [5, 6, 10–12] or dialectal variation [13], schwa maintains a more centralized position than all other English vowels in phonologically-similar environments [2, 10].

Schwa is also more vulnerable to coarticulatory effects caused by proximity to nearby segments [14–16]. For example, proximity to alveolar consonants has been shown to decrease schwa’s F1 formants and increase F2 formants, thereby resulting in higher vowels [2, 8]. Certain spectral qualities of schwa may also be more vulnerable to co-articulatory effects than others [8, 14]. This vulnerability has been attributed to under-specification of schwa for particular vowel quality contrasts, such as height or backness [8, 16–17]. Through gestural modelling of articulatory phonetics, Browman & Goldstein used nonsense word sequences to test whether schwa is unspecified for tongue position. Non-words containing schwa were mapped temporally and spatially as a series of gestural trajectories across syllable boundaries [8–9]. Ultimately, their gestural modelling indicated that schwa tends to gravitate toward a central vowel height despite proximity to anterior consonants—an indication that schwa is specified for at least the F1 acoustic dimension [8, 16–17]. Subsequent research on the under-specification of schwa has indicated that schwa may be unspecified only for the F2 (anterior/posterior) dimension, while centralized vowel height remains targeted across most speech [8, 15–17].

One phonological environment where more of schwa’s centralized vowel qualities are likely to be intact is in word-final position (e.g. “sofa” [sof**ə**], “Asia” [eʒ**ə**]) [12]. Word-final schwas have previously been identified as having more stable (i.e. reliable) F1 and F2 formant values than their word-internal counterparts [17–18]. Moreover, word-final schwa has been shown to maintain reliable F1 and F2 formants when it becomes word-internal due to the affixation of an inflectional morpheme such as the possessive {s} [17–18]. For example, Flemming & Johnson (2007) obtained more centralized formant values for schwa in “Rosa’s” [roz**ə**z] than in the similar construction “roses” [roz**ə**z] where schwa is phonologically-conditioned to occur only when the preceding morpheme ends in a sibilant. Though the second syllable of “Rosa’s” is closed due to affixation of the possessive morpheme, F1 and F2 spectral values were comparable to those in word-final position (i.e. open syllables) [17]. While others have found a three-way distinction in native English speakers’ F1/F2 formant values of schwa in the plural morpheme, before the possessive {s}, and word-finally, it does appear that word-final schwa and word-final schwa + possessive {s} belong to more robust categories of centralized vowels, whereas other types of syllable-final schwa have lower F1 values (corresponding to higher vowels) [18]. The discovery that schwa need not be entirely word-final to display stable spectral qualities supports our empirical assumption that word-final schwa in running speech can maintain centralized formant values due to its specification for certain dimensions in word-final position.

In addition to spectral qualities, phonological vowel reduction (not caused by superficial changes in speaking rate, amplitude, etc.) in English is characterized by shorter durations than those of stressed vowels [2, 6, 19]. As a general rule, reduced vowel durations are shorter word-internally than word-finally. However, schwas that are phonologically-conditioned for optional weak syllable deletion (e.g. between the stressed syllable and another syllable anchored by schwa) possess shorter mean durations than word-internal schwas not phonologically-primed for deletion [20–22]. Across all positions, schwa’s status as an unstressed vowel is highlighted by the large ratio of stressed vowel duration to unstressed vowel duration that characterizes English [6, 23–24].

The aforementioned studies examined the spectral and temporal qualities associated with vowel reduction by monolingual English speakers across a variety of phonologically-conditioned environments. Our study extends this line of investigation to explore the stability of spectral and temporal qualities of reduced vowels produced by early and late Spanish-English bilinguals living in the United States. For the purposes of this study, we adopt Grosjean’s (2010) succinct definition of *bilinguals* as “people who use two or more languages in their everyday life” [25]. While Spanish has traditionally been classified as a syllable-timed language, or one where syllable durations are of roughly equal length, lexical stress does create meaningful contrast [26]. Monolingual Spanish speakers’ ability to differentiate stress extends beyond meaningful contrasts, however, as evidence has suggested monolingual Spanish speakers are quite capable of differentiating non-words based on stress contrasts [27].

In speech production, certain monolingual varieties of Spanish tend to demonstrate slightly shorter vowel durations in unstressed syllables [28, 29]. However, centralization of unstressed vowels has found more empirical support in the speech of Spanish-English bilinguals than for monolingual Spanish speakers [30]. Moreover, atonic Spanish vowels have been observed to have more truncated durations and centralized spectral qualities in the Spanish of Spanish-English bilinguals than one typically records in monolingual Spanish [28–30]. For example, Spanish-English bilinguals living in the American Southwest have been shown to display greater centralization of unstressed vowels (i.e. lower F1 values) compared to monolingual Spanish speakers, notably in the case of /a/ raising to [ə] in atonic syllables [31].

Prior studies have also observed that both early and late Spanish-English bilinguals are capable of identifying English stress [21, 32–35]. That is, Spanish-English bilinguals have demonstrated sensitivity to the distributional patterns of English noun and verb stress that enable listeners to quickly classify real words by lexical category [33, 34]. However, neither early nor late Spanish-English bilinguals have reliably demonstrated speech output that approximates the difference between full and reduced vowel temporal qualities in accordance with the duration ratios that have been proposed for native-like American English speech rhythm [6, 36].

The underlying strategies used to produce stressed and unstressed English vowels may differ between English monolinguals and early or late bilinguals [32–35]. For early bilinguals, it may be the case that producing stressed and unstressed vowels with completely native-like temporal qualities is not necessary for intelligibility of speech. Hence, in some environments “compromise values” for duration may not impair intelligibility [18, 23]. This is more likely true in bilingual environments where the concept of “foreign accent” is gradient [30, 35]. For late bilinguals, on the other hand, non-native-like vowel reduction may be the result of transferring native-language (L1) Spanish stress rules to English [6]. For instance, Spanish stress has been shown to be tied to vowel duration over spectral qualities. As Spanish does not have vowels that contrast in both length and spectral qualities, as English does with its tense/lax distinctions, elongation of Spanish vowels serves only to enhance perceptual prominence (i.e. stress) [6, 23].

In many cases, singular attention to vowel duration will result in correct stress identification because increased duration, more peripheral spectral qualities, and prosodic enhancements (e.g. greater intensity) often co-occur [32, 37]. However, assigning stress to vowels based on duration alone can be problematic in languages that possess crowded vowel inventories, such as English, because differences in duration can be subtle and prone to variability. Vowel duration can be affected by neighboring segments, syllabic structure, or hyper/hypo-articulatory characteristics of the utterance [21, 35, 37]. While instances where intelligibility is compromised may be rare, over-reliance on one dimension of vowel stress can inhibit generalization of English stress assignment rules to new words.

Late Spanish-English bilinguals have demonstrated more success at assigning stress to English words when the target words are either frequently occurring or phonologically-similar to frequently occurring words [33, 35]. Though English has many rules for assigning and manipulating stress based on morphophonological alternations, the distributional frequency of various English syllable structures appears to influence the likelihood that Spanish-English bilinguals will correctly assign stress [6, 33, 35]. For instance, late bilinguals quickly master the stress-shifting rule that disyllabic nouns typically stress the penult syllable whereas verbs place stress on the ult, and are therefore able to contrast between the noun “IMport” and the verb “imPORT” without difficulty [21, 34]. However, words that have atypical syllable structures or occur in sparse phonological neighborhoods typically receive less accurate stress placement by late bilinguals [33, 35]. Guion, Harada, and Clark (2004) observed the effect of lexical category on accurate stress placement was also less robust for late bilinguals than for early bilinguals [35]. For example, early Spanish-English bilinguals produced L2 English words containing either marked or unmarked syllable structures with nearly native-like stress placement, with the exception of observed tendencies to avoid word-final vowel lengthening [35]. Late bilinguals, on the other hand, deviated more abruptly from correct English stress placement when the phonotactic probability of the syllable structure decreased [33, 35].

Issues concerning late bilinguals’ ability to accurately assign stress are significant in light of prior claims that accurate stress placement chronologically precedes accurate vowel reduction in speech production [21–23]. However, stress assignment is just one reason that spectral quality and temporal deviations from monolingual English speech can occur. Another potential contributive factor is lack of sensitivity to phonetic or prosodic features of the L2 that are not contrastive in the bilingual’s native language [21, 32]. In Spanish, there is no *phonemic* contrast for vowel duration [30]. English, on the other hand, does contrast vowels by duration under the tense/lax vowel distinction [38]. Therefore, maintaining categorical separation of target vowel durations is important for maintaining lexical contrast [38]. In general, Spanish-English bilinguals produce longer vowel durations in Spanish than their monolingual counterparts as well as shorter ratios of stressed-unstressed syllable durations than monolingual English speakers [28]. In addition to these sorts of “compromise values,” vowel durations in atonic syllables vary more widely for Spanish-English bilinguals than English monolinguals, which may indicate prosodic targetlessness that persists after segmental mismatches have been resolved [28].

Therefore, mismatches between the role of vowel duration in the L1 and L2 of Spanish-English bilinguals may contribute to longer unstressed vowels as well as smaller ratios for the duration of stressed-to-unstressed vowels than monolingual speakers typically produce.

As language learning is refined over the lifetime [39], early bilinguals’ prolonged experience perceiving and producing L2 English may result in more native-like L2 phonetic output [40]. Over time, L2 speakers may perceptually attune to differences between their articulatory output and phonetic properties of the L2. Heightened sensitivity to subtle differences between a bilingual’s L1 and L2 phonologies may stem from more exposure to native L2 speakers, more practice speaking in his/her L2, or explicit pronunciation feedback [41, 42]. This process is outlined by Flege’s Speech Learning Model and its subsequent revisions [39–41, 43–44]. Early in the L2 acquisition process, it is anticipated that speakers whose L1 contains neither central vowels nor mandatory stress assignment will substitute peripheral vowels in place of schwa [21, 39–41]. This tendency is magnified in any sort of reading task, orthographic representations of unstressed vowels correspond to peripheral vowels [44]. Refinement of unstressed vowel category boundaries (i.e. schwas) occur as a result of increased exposure to L2 speakers, more practice using the L2, or through explicit instruction such as contrastive analysis of L1/L2 phonetic properties [40–42]. Of these factors, none has attracted as much attention, or been contested as bitterly, as age of L2 learning [44–51].

While the presence of a foreign accent is likely the result of several factors working in unison, there is evidence that ‘age of L2 learning’ is one of the most reliable contributors to native-like L2 phonetic production [47–49]. A half-century ago, Lenneberg argued that there is a “critical period” which exists for language learning [50]. The idea that one’s opportunity for native-like L2 phonological acquisition closes after a certain biological window continued to gain support over subsequent decades [43, 45, 47, 51]. Patkowski described the critical period for L2 phonological acquisition as “… a period, ending around the time of puberty, during which it is possible, but not inevitable, for L2 speakers to acquire as an end-product of a naturalistic L2 acquisition process full native-like fluency in the phonological system of a second language, and after which such a possibility does not exist anymore” (see p. 206 of [47]). Studies have also observed a gradual decline in native-like L2 phonetic output as age of L2 learning increases [36, 38, 46, 52]. However, there does not appear to be either a biological impetus for decreased phonetic accuracy or an abrupt closing of the window where pre- and post-critical period boundaries can be delineated [10, 39, 48, 49].

The question of why L2 pronunciation appears to be hindered by advanced age of L2 acquisition when other language areas (such as vocabulary and syntax) do not has been offered mainly perceptually-based explanations [32, 54]. People who began learning their second language in adulthood may be disadvantaged when learning new phonetic categories because their L1 linguistic experience has accustomed them to grouping perceptually similar sounds as manifestations of the same phoneme [39, 40]. To produce native-like L2 sounds, late bilinguals must reshape their categorical boundaries based on L1 phonology to accommodate incoming L2 phones [40, 43, 54, 55]. To date, few people have suggested that even early bilinguals (who continue to use both languages) speak each language free from any cross-linguistic interference [10, 56, 57]. Studies that seem to contradict this statement have highlighted cases where bilinguals “pass” as native speakers; however, these case studies typically involved speakers of typologically-related languages (e.g. two Germanic languages) or accent judges who were unfamiliar with the phonetic features of certain “foreign accents” [56–58].

In recent years there has been resistance to the idea that late bilinguals face inevitable limitations on their capacity to achieve native-like L2 pronunciation [58–60]. A major criticism has been that, to date, there is not neurophysiological evidence of a causal relationship between cerebral maturation and inability to perceive and produce L2 sounds with native-like pronunciation [60–64]. Suggestions regarding loss of “neuroplasticity” in the brain [47] suggest that certain connective pathways (i.e. “white matter”) between phonological processing and motor speech planning regions do not form easily after the brain has reached maturity. Exactly how language learning differs from other types of learning in the brain has not been established to date [48]. For instance, studies of external neurological activity (electroencephalography) during classroom-style language instruction have indicated changes in electrical patterns in brain activity during the earliest stages of L2 acquisition [62]. In addition, some patients with aphasia (a neurological disorder characterized by difficulties using language) have experienced functional reorganization of connective pathways in their brains to circumvent damaged pathways following a cerebrovascular accident [62–64]. This discovery at the very least suggests that neuroplasticity is not controlled by exclusively biological factors [65]. In summary, physiological evidence of neuroplasticity after puberty indicates two important points regarding age and phonological learning: First, claims that a biological window that closes upon the opportunity to attain native-like pronunciation have yet to be concretely substantiated at either structural or functional levels of neural organization [61]. Second, we have learned from post-stroke patients that the brain is capable of reorganizing large-scale systems, including those for processing and producing language [63].

If reduced ability to accurately acquire L2 phonologies after puberty is not entirely biologically-driven, then the most likely explanation is that it is caused by either cognitive or experiential factors, or a combination of two. In recent years, cognitive models of working memory and information processing have been modified to incorporate the role of language and sub-vocal rehearsal in maintaining auditory information [65–66]. If we adopt the assumption that language acquisition is a form of information processing, then the native language necessarily creates a filter through which new languages are processed and organized [65, 67]. Cognitive abilities that can impact native-like L2 speech production include executive functions as pattern recognition, selective attention, and task switching [65, 67, 68]. A review of the role individual cognitive abilities play in L2 vowel production is beyond the scope of the current article; however, Abutalebi & Green (2007) provide a comprehensive review of the relationship between bilingualism, executive control, and functional imaging of language tasks in the brain [68].

Though age of L2 learning may be a comparatively reliable predictor of the likelihood of acquiring a native-like L2 phonology, experiential factors also play a role in predicting the outcome. Experiential factors include amount of L1 use [25, 57, 69–71], formal education in one or both languages [20, 72], and the native-speaker status of the L2 community [69]. These factors may overlap to varying degrees with nonstructural features such as linguistic attitudes [73], ethnic identity of the L2 speaker [74], and motivation to achieve native-like pronunciation in the L2 [75]. Of the experiential factors listed, one that has been shown to negatively predict native-like L2 speech production is amount of L1 use [57]. Using one’s native language more often is believed to strengthen its level of activation, thereby creating more opportunities for phonetic interference than one predicts in bilinguals who seldom use their native language [25]. For example, Italian-English bilinguals living in Canada who often speak Italian have been judged by monolingual English speakers to possess stronger foreign accents than their age-matched peers who speak Italian less often [41]. Children who spend a greater proportion of each day speaking in their L2 typically reflect less accented L2 pronunciation [71], as do adults who work in L2-immersive environments [57]. Amount of L1 use often overlaps with age of L2 learning because early bilinguals often have spent much of their day being educated in their L2 and interacting with L2-speaking peers—two avenues that have been suggested to motivate more native-like L2 speech output [53, 73–76].

Late bilinguals, on the other hand, have received at least some of their education in their native language. If this education included literacy training, as is likely the case, then late bilinguals likely possess some degree of L1 orthographic interference when reading L2 words. Only if a late bilingual is immersed in L2 writing and media for an extended period would we expect orthographic effects to diminish. As previously mentioned, interacting with peers is another source of motivation for improving L2 pronunciation. Late bilinguals may be less motivated to develop native-like L2 speech for a variety of reasons. For instance, native-like L2 pronunciation may conflict with a bilingual’s ethnic identity [74]. Late bilinguals are also more likely to have established relationships with people who share their native language, thereby dampening their motivation to approximate the speech of monolinguals who speak their L2 [75, 76]. Differences notwithstanding, even late bilinguals may alter their phonetic output as phonemic category boundaries change with experience [32, 39, 40]. Furthermore, “gestural drift” can occur in both early and late bilinguals when they move between monolingual L1 and monolingual L2 environments [70].

Various linguistic history questionnaires have probed bilinguals’ attitudes toward their languages using a variety of objective and subjective questions. Some questionnaires focus heavily on the amount of time engaging in L1 vs. L2 use and draw distinction between time engaging in expressive versus receptive language use [77–79]. Others have aimed to capture subjective measures, such as the level of importance the individual assigns to a language and the emotional attachment he/she feels toward one language versus the other [80]. Taken together, these measures contribute to the factor “bilingual dominance” that can predict how native-like we expect L1 and L2 speech output to be [80]. Bilingual dominance scores that also include subjective measures of linguistic attitudes can be instrumental in explaining why phonetic output varies within communities that are relatively homogenous according to many sociolinguistic assessments.

The current study examined vowel reduction in word-final position among groups of early and late Spanish-English bilinguals, as well as English monolinguals. To create a balanced stimuli set that accounts for possible co-articulatory effects due to the preceding consonant, many of our target words are cognates for Spanish and English. Cognates have previously been identified as more vulnerable to phonetic interference compared to non-cognates [80]. However, the direction of transfer is not unidirectional L1→L2 as one would predict if L1 literacy effects were the underlying cause. Amengual (2012) observed that Spanish-English bilinguals produce longer voice onset times (VOTs) for cognates than for non-cognates [80]. However, it has been noted that VOTs for /t/ are particularly unstable in Spanish-English bilinguals [56], and this segment showed the most cross-linguistic interference among voiceless stops [80]. Spanish-English bilinguals have also demonstrated clearer categorical separation of VOT values for cognates pronounced in Spanish and in English when stimuli were presented entirely in English or Spanish, compared to code-switching constructions [56, 81]. These findings suggest that placing a bilingual in a monolingual language mode can suppress cross-linguistic phonetic interference for bilinguals (at least those who are highly literate in the language being tested) even if the target words have cognate status.

All of our participants were residents of Miami, Florida who were enrolled as students at Florida International University, where courses are taught in English. Extensive experience with L2 reading/writing as well as monolingual L2 English language mode in the classroom (due to English-only instruction) positions both early and late bilinguals to produce English sentences with diminished L1 orthographic transfer. Our study further examined how well three linguistic history factors predicted native-like L2 vowel reduction. These factors were: Age of L2 learning, amount of L1 use, and self-reported bilingual dominance scores. To date, there are no studies establishing early and late bilinguals’ average schwa durations and vowel qualities in word-final position, where centralization of the unstressed vowel is more likely to be maintained than word-internal atonic syllables [2, 17]. Detailing vowel reduction patterns in these three groups of Miami-based speakers will also contribute valuable phonetic data to ongoing efforts by sociolinguists to categorize an emerging “Miami accent” [82].

We predicted that late bilinguals would vary their word-final schwa vowel qualities more than monolinguals and early bilinguals due to orthographic interference effects during our reading task. We also expected more evidence of an [a]/[ə] merger in late bilinguals’ speech from orthographic effects, while early bilinguals were predicted to produce compromise vowel qualities half-way between monolingual English speakers’ and late bilinguals’ vowel qualities due to having both languages heavily activated in the heavily bilingual city of Miami. In addition, we predicted longer word-final schwa durations for late bilinguals because they are expected to display smaller ratios for the duration of stressed and unstressed vowels compared to early bilinguals and monolinguals. Early bilinguals were predicted to produce longer vowel durations than monolingual English speakers, but shorter than late bilinguals, because interactions within their L1/L2 system may result in compromise values for all centralized vowels that are longer than [ə], but shorter than [a]. We did not predict that orthographic interference would affect our early bilinguals because most of them were not educated in Spanish beyond what any high school student receives, regardless of language background.

Before moving on, it is important to acknowledge that Miami-based English monolinguals may not produce the same vowel qualities and reduced vowel durations that one expects from general American English. For instance, English monolinguals living in Miami have recently been shown to deviate prosodically from general American English. Early characterizations of Miami-accented monolingual English include reduced duration ratios between stressed and unstressed syllables (giving the impression of a more syllable-timed English) as well as subtler variation in pitch for stressed syllables [83, 84]. However, we maintain that Miami English should be considered the “target English,” despite access to general American English in the media, because it is the source of L2 input and spoken across social classes without stigma.

We further predicted that within each bilingual language group, more native-like reduced vowel production could be predicted by the two linguistic factors “amount of L1 use” and “bilingual dominance score.” We were aiming to determine if certain factors predicted either vowel qualities, vowel duration, or both. Additionally, we sought to discover whether different factors predicted native-like schwa production in early bilinguals than in late bilinguals. Specifically, we predicted that speakers whose scores indicated more motivation to retain and improve their L2 would produce more native-like schwas than those who maintained a stronger social and emotional connection to their native language. We ranked the explained variance of our nonstructural variables differently for duration than for vowel qualities, which fulfilled our aim of exploring how nonstructural variables predict phonetic variation in bilingual speech.

## Methods

This experiment was designed to test early and late bilinguals’ production of schwa in word-final position elicited from a reading task. It was approved by the Florida International University Review Board as IRB-011212-02-TR “Variation in the Production of Reduced Vowels by Monolingual and Bilingual Populations” as one of several proposed studies examining segmental variation. We aimed to determine if the two bilingual groups systematically produce different vowel durations and/or spectral qualities from Miami’s monolingual English speakers [17, 20]. We also examined three potential predictors of native-like L2 phonetic output including age of L2 learning, amount of L1 use, and bilingual dominance scores.

### Participants

Our subjects consisted of students enrolled at Florida International University—a public university in Miami, Florida. This campus is representative of the demographic makeup of the city of Miami. That is, 61% of the student body identifies as Hispanic, 15% as white non-Hispanic, 13% as black non-Hispanic, 4% as Asian, and 7% as “other” [85]. Spanish is the second most widely spoken language and it is commonly spoken across campus by both students and employees. With few exceptions, courses are taught in English, thus ensuring that all subjects were literate and orally proficient in English. Subjects were recruited from undergraduate and graduate phonetics and speech pathology courses and were compensated with minimal extra credit for their participation.

Our subjects were assigned to one of three language groups: monolingual English, early Spanish-English bilingual, or late Spanish-English bilingual. Monolingual English speakers functioned as controls for the acoustic-phonetic parameters that we could expect to early and late bilinguals to target. We were open to the possibility that Miami-based monolinguals would have phonetic features that deviated from “typical” monolingual American English vowel qualities and durations as traditionally described [2, 5, 8, 12, 17, 19, 86]. Our monolingual English speaker group consisted of 20 subjects (8 males, 12 females) whose ages ranged from 18–33 years old (*M* = 24.4). Monolinguals had lived in one of the more heavily English-dominant neighborhoods/townships of Miami (e.g. Coconut Grove or Coral Gables) or in the predominantly non-Hispanic area of Aventura between Miami and Fort Lauderdale [87]. All subjects were required to have lived in Miami for a minimum of five years (*M* = 7 years) and they self-reported that they engage in daily interactions with Spanish-English bilinguals, either at work or in the community.

Monolingual English subjects were not included in the study if their speech reflected noticeable dialectal markers (e.g. features of New York/Jewish accent or African American Vernacular English.) To confirm that monolinguals sounded “local,” we asked two Miami-born Spanish-English simultaneous bilinguals [88] to listen to clips of the monolingual speech and rate the likelihood that the speaker is “from Miami” on a 1–7 scale, where one was “definitely from Miami” and 7 was “definitely not from Miami.” Our accent judges were asked to supply the geographic region they believe the speaker to be from if they rated his/her speech over a 5. The purpose behind this modified Likert Scale task [89] was to establish that the phonetic output of our monolingual speakers was the same as the input Spanish-English bilinguals receive from monolinguals in the ambient community.

Our English monolingual subjects (EMs) reported that they spoke no other language beyond typical exposure in high school courses and that they had not extensively travelled or lived abroad. To objectively confirm each subject’s status as an English monolingual, he/she was given a brief test of receptive Spanish vocabulary knowledge using the Vocabulario en Imágenes Peabody [90] adapted from the more commonly-administered Peabody Picture Vocabulary Test in English [91]. No EM scored beyond the basal set of stimuli on the Spanish-language TVIP. Lastly, our subjects reported no known cognitive or attention deficits or learning disabilities of any kind. They were instructed to wear corrective lenses if prescribed. Hearing was not assessed because our experiment was a non-auditory reading task.

Our bilingual groups were divided into two equal groups of early and late bilingual speakers. Early bilinguals (*M* = 20; 12 males and 8 females) ranged in age from 18–31 years (*M* = 21.1). Early bilinguals were born in the following countries or territories: United States (FL/NYC) (13), Cuba (3), Puerto Rico (2), Honduras (1), Venezuela (1), Dominican Republic (1). The criteria for “early bilingual” were that subjects began learning L2 English between the ages of birth and eight years. These parameters are conservative in that they are far-removed from onset of puberty [47–50, 60, 92]. Many EBs self-reported beginning to learn L2 English in preschool or kindergarten, having spoken primarily Spanish in the home with their caretaker until they went to school (Age of L2 acquisition (AoA) range = 0–7 years; *M* = 2.9 years). However, some EBs with older siblings reported limited English proficiency, or the ability to code-switch, from the beginning of his/her language acquisition. All early bilinguals reported that Spanish continues to be spoken in the home, albeit to varying degrees.

Our late bilingual (LB) subjects acquired English no sooner than age 15, several years past the “onset of puberty” divide [47–50, 92]. The late bilingual group (*N* = 20; 7 males and 13 females) ranged in age from 23–40 (*M* = 31.1). Late bilinguals’ age of L2 acquisition ranged from 15–24 years of age (*M* = 18.3 years). In general, late bilinguals living in Miami are born in South America, Central America, or the Caribbean and come to Miami during adolescence or early adulthood. LBs in this study were born in the following countries (or territories): Cuba (9), Venezuela (3), Dominican Republic (2), Colombia (2), Argentina (2), and Puerto Rico (2). The main reasons LBs gave for moving to the United States were to attend school (secondary or university), to live with family who were already settled in South Florida, and for marriage. All LBs enrolled in American universities are required to pass the Test of English as a Foreign Language (TOEFL) [93] to demonstrate written and oral proficiency in English. As with the early bilinguals, late bilinguals reported daily use of Spanish in addition to their English-only class time. Three late bilinguals were not selected for the study due to self-reported advanced proficiency in a third language, while an additional bilingual was excluded due to having an age of L2 learning that did not meet the criteria of either bilingual group.

### Linguistic assessments

Prior to inclusion in the study, all participants were given an initial oral interview (in English) where each person described his/her language experience in their own words. Subjects were then given consent forms to complete, as well as a copy of the *Language Experience and Proficiency Questionnaire* to assess age of L2 learning and quantify amount of L1 use [94]. In addition, early and late bilinguals also provided qualitative and quantitative views on their language use via a bilingual dominance assessment [95] so that a composite bilingual dominance score could be obtained for each subject. Questions from the bilingual dominance assessment included quantifiable measures, such as the number of hours per day spent reading/speaking/listening to media in each language. In addition, the bilingual dominance assessment considered subjective language attitudes. For example, the assessment asks bilinguals which language they would keep if they could only keep one. Possible scores ranged from -30 to 30, where -30 indicates a monolingual who doesn’t speak the ambient language (in this case a Spanish monolingual) and 30 indicates a speaker who is monolingual in the ambient language (i.e. English monolingual). A score of zero indicates a completely balanced bilingual. To qualify for the study, all participants were required to have bilingual dominance scores between -10 and 10.

### Stimuli

To examine schwa duration and vowel qualities between our language groups, an English-language reading task was created. Fifteen of the most frequently-occurring disyllabic words containing word-final schwa were selected as target words for the experiment [96]. The words we selected had also been previously rated as “highly familiar” by monolingual English-speaking students [97]. Target words were divided into three categories based on the point of articulation of the preceding consonant. These categories included: post-labial schwa, post-coronal schwa, and post-dorsal schwa. It is worth noting that a discrepancy exists between the point of articulation for English and Spanish stops /t,d/. English /t,d/ are typically located at the alveolar ridge (with dental and interdental allophones in certain phonological environments) [98, 99], whereas Spanish /t,d/ are typically produced at the back of the superior incisors [100]. For the purposes of this study, we emphasize that the three categories of preceding consonant that we have identified correspond to labial, anterior, and posterior sounds in both languages.

Each target word was embedded into the initial determiner phrase of each sentence in order to maintain similar prosodic rhythms and levels of focus across sentences. In addition, each target word was followed by an alveolar stop to avoid regressive co-articulatory effects brought on by anticipation of the following consonant. In addition to the target sentences, filler sentences of varying length and intonation patterns were added to the stimuli to prevent speaker boredom. These sentences did not contain any word-final schwas. Examples of filler sentences are “We’ll probably go shopping tomorrow” and “Don’t tell mom we’re leaving on Sunday.” Sentences were loaded into a PowerPoint presentation where one only sentence appeared per slide. Sentences are listed in Table 1.

**Table 1:** Sentences containing word-final schwa targets. The “post-labial” category includes schwas that follow [m], [b], and [v]. “Post-coronal schwas” follow [s], [ʒ], [z], [ʤ] and [ʃ]. “Post-dorsal” schwas follow [k] and [g].

Post-labial schwa	Post-coronal schwa	Post-velar schwa
A loofah [lufə] takes off dead skin.	Touring Russia [rʌʃə] takes time.	Use a hookah [hukə] to take in smoke.
A dogma [dɑgmə] takes faith to accept.	A ninja [nɪnʤə] tiptoes in the dark.	Hot mocha [mokə] tastes better than tea.
A comma [kamə] tells the reader to pause.	Marsha [mɑrʃə] takes piano lessons.	Crushed mica [maɪkə] twinkles in the sun.
The tuba [tubə] takes strength to play.	Lisa [lisə] talks too much.	Try yoga [jogə] to stretch your body.
Hot lava [lɑvə] takes time to cool.	Asia [eʒə] tries to preserve its history.	That saga [sɑgə] took hours to retell.

### Procedure

Subjects were interviewed by the researchers in English before the experiment. During this time, they completed consent forms, language history questionnaires, receptive vocabulary tests in English and Spanish, and bilingual dominance assessments as described earlier. All assessments except the TVIP were conducted in English. After the assessments, subjects were given a ten-minute break but asked to remain in the linguistics laboratory. At the conclusion of the break, subjects were escorted into a sound-proof room and seated in front of a Dell XPS 13 Ultrabook laptop. Participants were told that the study involved reading sentences on a computer screen and that their speech would be recorded and later analyzed. They were instructed to read the sentences at a natural, conversational pace and not to go back and correct mistakes. While subjects were able to self-advance the sentences by pressing the down arrow key, they were also informed that sentences would “time out” after five seconds. Subjects were further instructed that if they did not complete a sentence before it timed out, they should go on to reading the next sentence. This time-out method was implemented to maintain conversational speed in subjects’ reading and reduce hyper-articulatory effects [37]. As our target words occurred in the opening determiner phrase, tokens were not lost due to incomplete sentences.

At the beginning of the experiment the subject was prompted by the researcher to read the sentence aloud, followed by two more familiarization trials with the researcher present. Recordings were made using a SONY-PCM D50 portable digital recorder with a sampling rate of 44.1 kHz. Each subject’s output was automatically stored as a.wav file and later spliced into individual sentence.wav files by the researcher using PRAAT speech analyzing software [101]. At the end of the three familiarization trials, the researcher checked the recorder to ensure it was working and the speaker had adequate volume on the recordings. A new file was then started for the experiment. The researcher told the subject to push “enter” when ready to begin the task and the subject was left alone to read the sentences aloud. Each subject completed the task in approximately 15–20 minutes.

### Measurements

Target schwas were isolated from the speech stream using the chop and label method available in PRAAT [101]. To segment word-final schwa from the preceding consonant, we first located the constriction offset using such spectrographic features as voice onset time (VOT) for voiceless stops, termination of the voice bar for voiced stops, or fricative noise release for fricatives and affricates [6]. For example, “soda” would begin after the release of the [d] closure, typically characterized by the offset of the voice bar [6, 10]. The onset of the vowel was identified as the onset of periodicity (defined by clear formant structure in the spectrogram) following the acoustic cues. Increases in the amplitude of the waveform were taken as secondary cues for the onset of schwa [28]. We obtained values for three acoustic measurements: duration, F1 and F2 frequencies. Automated PRAAT scripts extracted F1 and F2 values in Hz at the midpoint of each target schwa, while a second PRAAT script automatically measured the duration of target schwa in milliseconds [102]. In addition, F2-F1 values were calculated from these measurements using Microsoft Excel. Formant values and durations were later checked by a second researcher. Agreement constituted +/- 3 milliseconds for duration and +/-15 Hz for F1 and F2. Inter-judge reliability was 93% for duration and 97% for F1/F2 formant values.

### Analysis

A multivariate analysis of variance (MANOVA) was created in SPSS version 24 using raw values for three dependent variables: F1, F2, and the difference found by subtracting F2-F1. F2-F1 values serve as an indicator of a vowel’s corresponding position in the oral cavity along the vertical and anterior-posterior dimensions [103–105]. Acoustic qualities of vowels are affected more by the gender of the speaker at the F0 level than at higher F1 and F2 formant bandwidths [105]. Moreover, vowel normalization procedures have been less effectively applied to multiple formant frequencies than to altering one single frequency (e.g. F1) for a vowel [106].

We performed a one-way MANOVA that considered our spectral measures as a group of related dependent variables. Our independent variables (or fixed factors) were the place feature of the preceding consonant (labial, coronal, or dorsal) and language group (EM, EB, LB). “Individual” (subject) and sentence were added as covariates to control for individual variation among subjects and/or variable accuracy pronouncing target words. In addition, a separate univariate analysis of variance (ANOVA) was constructed along these same parameters identifying “duration” as the single dependent variable. Additionally, a multivariate multiple regression was performed using the general linear model to examine whether any of our nonstructural variables predicted native-like L2 vowel reduction. Our dependent measures included all spectral quality measures (F1, F2, F2-F1) and schwa duration. Predictors added to the model were our nonstructural variables: age of L2 learning, amount of L1 use, and bilingual dominance score (BDS).

## Results

### Spectral qualities

Prior to synthesizing the results of the models, descriptive statistics for the mean formant values and duration of schwa following labial, coronal, and dorsal consonants are presented in Table 2. Values reflect the estimated marginal means taken from a one-way multivariate analysis of variance (MANOVA) model, rather than raw means [28].

**Table 2:** Duration and vowel quality means^a^ for word-final schwa produced by English monolingual, early Spanish-English bilinguals, and late Spanish-English bilinguals.

	Place of preceding consonant	Number of cases	F1 (Hz)	F2 (Hz)	F2-F1 (Hz)	Duration (seconds)
**EM**	Labial	100	493.85	1634.58	1140.73	.073
	Coronal	100	477.18	1859.70	1382.52	.075
	Dorsal	100	520.92	1737.73	1216.81	.095
**EB**	Labial	100	509.76	1620.65	1110.89	.088
	Coronal	100	481.36	1782.19	1305.38	.074
	Dorsal	100	519.19	1633.26	1114.07	.091
**LB**	Labial	100	613.75	1436.42	822.67	.117
	Coronal	100	587.19	1691.20	1104.01	.104
	Dorsal	100	606.53	1508.29	901.76	.137

^a^ Values are estimated marginal means, not raw averages

**Fig 1:**
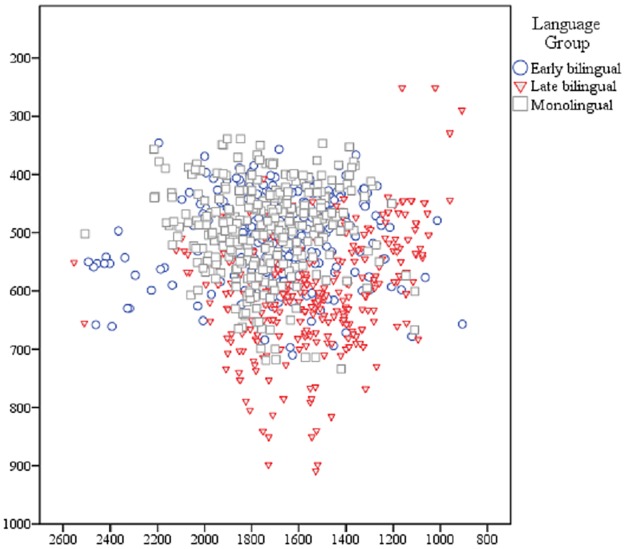
Raw spectral values of schwa for all language groups. The x-axis shows F2 formants that correspond to a vowel’s position along the anterior-posterior dimension of the oral cavity. The y-axis shows F1 formants that correspond to vowel height.

Multivariate results indicated significant main effects for both independent variables “language group” (*F* (4, 1776) = 33.56, *p* <.001) and “preceding consonant” (*F* (6, 1776) = 10.83, *p* <.001). The interaction of language group * preceding consonant was not significant, *p* = .235. Results further indicated that a sizeable portion of the overall variance in vowel qualities that was present for variables F1, F2, and F2-F1 was explained by the predictors language group and preceding consonant (Adjusted *R*^*2*^ = .400). Language group and preceding consonant were also significant predictors for each of the spectral variables when considered individually, *p* <.001. Within groups, results indicated that schwas were systematically influenced by the phonetic features of the preceding consonant for at least one formant. Of the individual spectral variables, language group explained the highest percentage of variance for F1 (η^2^ = .11) with considerably less variance explained for F2 or F2-F1 (η^2^ = .03 for both DVs). In general, LBs produced higher F1 values than either EMs or EBs, indicating a lower vowel position. EMs demonstrated the most homogenous spectral qualities for word-final schwa, while EBs differed markedly in their F2 values (Fig 1).

Pairwise comparisons further revealed that not every group differed significantly from the other two along every spectral measure. For example, F2-F1 values (overall vowel position in the oral cavity) indicated that EMs produce schwa at points of articulation that differ from either EBs (*p* <.001) or LBs (*p* = .019), whereas the difference between EBs and LBs failed to reach significance, *p* = .544. F1 differences between EMs and EBs were also less robust, although significant, *p* = .024 [Table 3].

**Table 3:** Pairwise comparisons of F2-F1 formant values for English monolingual, early Spanish-English bilinguals, and late Spanish-English bilinguals.

Dependent Variable	Group	Group	Mean Difference	S.E.	Sig.	95% C.I.	
						Lower	Upper
F2-F1 Hz	EB	LB	18.79	30.95	.544	-41.96	79.54
		EM	145.26*	30.95	.000	84.51	206.02
	LB	EB	18.79	30.95	.544	-79.54	41.96
		EM	126.47*	53.63	.019	21.21	231.74
F1 Hz	EB	LB	120.54*	12.23	.000	-144.55	-96.53
		EM	27.61*	12.23	.024	3.60	51.62
	LB	EB	120.54*	12.23	.000	96.53	144.55
		EM	148.15*	21.19	.000	106.56	189.75
F2 Hz	EB	LB	102.20*	31.91	.001	-164.85	-39.56
		EM	170.30*	31.91	.000	107.65	232.94
	LB	EB	102.20*	31.91	.001	39.56	164.85
		EM	272.50*	55.30	.000	163.97	381.04

Comparisons between English monolinguals (EM), early bilinguals (EB), and late bilinguals (LB) are based on estimated marginal means.

* The mean difference is significant at the .05 level

### Duration

For duration, there was a significant main effect of language group (*F*(2, 891) = 82.46, *p* <.001). However, this effect was not equal across all groups. Pairwise comparisons revealed that LBs had significant duration differences compared to EMs (*p* <.001) and EBs (*p* <.001). EBs and EMs did not differ significantly with regard to duration, *p* = .401.

A second significant main effect of preceding consonant on schwa duration (*F*(2,891) = 25.45, *p* <.001) was found. Specifically, a lack of significant difference between EM and EB schwa production was affected by the occurrence of certain preceding consonants. In addition, the interaction between language group * preceding consonant was significant, *p* = .029. For EMs, mean schwa values were nearly identical when following either of the anterior consonants (i.e. labial and coronal), as summarized in Table 2. EBs and LBs, on the other hand, had mean schwa durations that categorically differed according to the preceding consonant. Post-labial schwa had the most separation between the groups.

**Fig 2:**
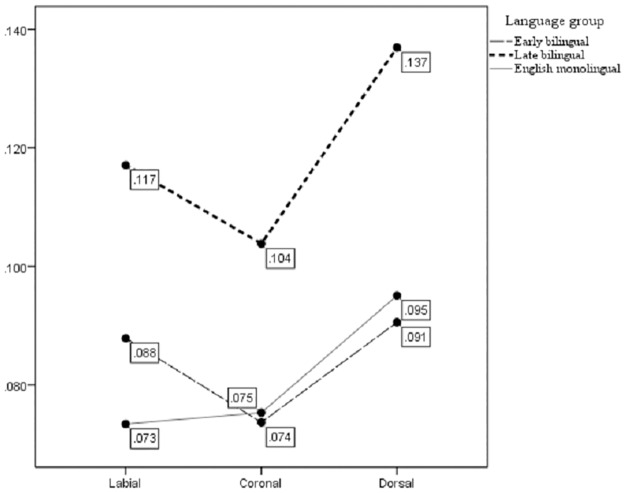
Mean durations for schwa by preceding consonant. The X-axis identifies the place of articulation of each category of consonant that precedes schwa. The Y-axis shows duration in seconds. Each line represents a different language group.

In general, EMs and EBs produced schwa in post-coronal and post-dorsal position with nearly identical durations. The pattern can be summarized as follows: EMs produced post-labial and post-coronal schwas with nearly equal durations, while EBs produced post-labial and post-dorsal schwa with nearly equal durations. LBs, on the other hand, exhibited wide differences between the duration of schwa in post-labial, post-dorsal, and post-coronal positions. Across all preceding consonant categories, LBs produced schwa following all three places of articulation with longer durations than those observed for either EMs or EBs. As Fig 2 illustrates, both bilingual groups adhere to the following durational hierarchy for schwa: post-coronal < post-labial < post dorsal. English monolinguals do not contradict this pattern; rather, their overall shorter schwa durations have less range for categorical separation of durations following anterior consonants.

### Nonstructural measures

As mentioned above, we explored the role three nonstructural variables had in predicting native-like vowel reduction. These variables were age of L2 learning, bilingual dominance score (BDS), and amount of L1 use. While it is commonly accepted that age of L2 learning is a solid predictor of native-like L2 production (although not a guarantee), the other role played by bilingual dominance and amount of L1 use is less clear. We performed two multiple multivariate linear step-wise regressions to explore whether these three nonstructural measures explained the variance in schwa duration for EB and LB groups. One multiple regression was performed for duration and the other to predict the variance for spectral qualities of schwa.

#### Nonstructural predictors of schwa duration

**Fig 3:**
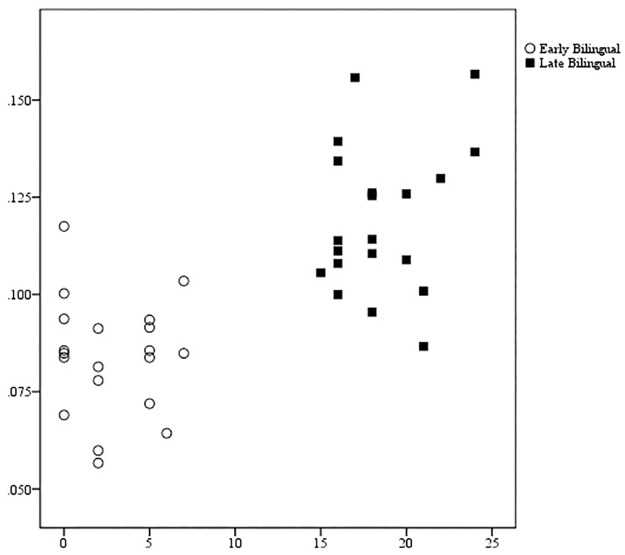
Age of second language learning predicts word-final schwa duration. The x-axis shows the age when second language learning began (in years). The y-axis shows the duration of word-final schwa in seconds.

Taken together, the three predictors (AoA, amout of L1 use, and bilingual dominance score) explained a sizeable portion of the variance for schwa duration (*R*^2^ = .54, *p* <.001). Of the three predictors, age of L2 learning was the greatest predictor of word-final schwa duration (*R*^2^ = .51, *F*(1, 39) = 39.27, *p* <.001). Amount of L1 use had a marginally significant main effect *F*(1, 39) = 3.26, *p* = .08, *R*^2^ = .08. Bilingual dominance score was not a significant predictor of schwa duration. Essentially, as age of L2 learning increased, word-final schwa duration also increased (Fig 3). However, the individual data points in Fig 3 also show considerable overlap between certain EB and LB speakers for word-final schwa duration.

**Fig 4:**
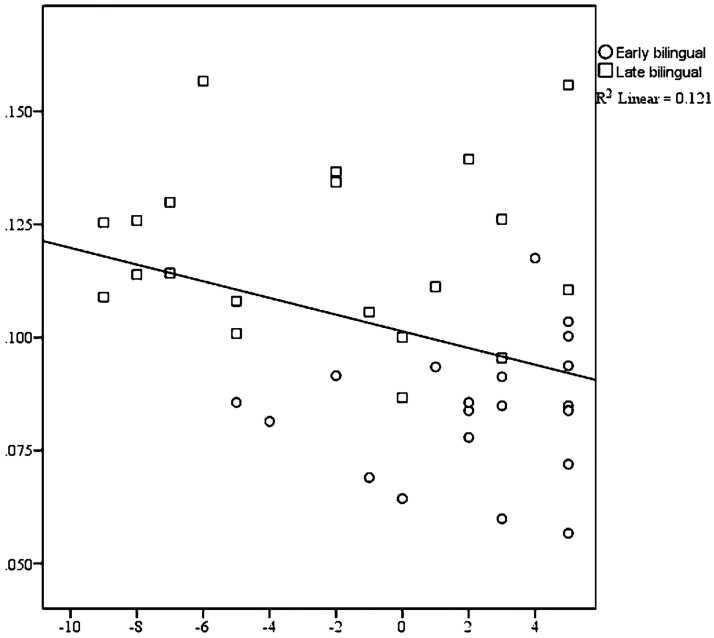
Early age of L2 learning predicts higher bilingual dominance scores and lower word-final schwa durations. The x-axis shows the range of reported bilingual dominance scores, where negative scores indicate L1 dominance and positive scores indicate L2 dominance. The y-axis shows word-final schwa durations in seconds.

Bilingual dominance scores were the other significant predictor of the variance in word-final schwa durations (adjusted *R*^2^ = .10, *F* (1,38) = 5.25, *p* = .028). Recall that bilingual dominance scores (BDS) were calculated on a scale of -30 to 30, where -30 is an L1 heritage language monolingual (in this case Spanish), 30 is a dominant language monolingual (English), and zero is a completely balanced bilingual [79]. Therefore, any subject who has a negative BDS prefers to speak, or is dominant in, L1 Spanish. Any subject who has a positive BDS prefers to speak English or is dominant in that language. It should be noted that separating subjective linguistic preference from empirically-validated linguistic dominance is beyond the scope of the current study. Results from the regression indicate a negative relationship between duration and L2 dominance—as bilingual dominance shifts toward L1, mean schwa durations increase. EBs demonstrated more within-group homogeneity than LBs for this measure (Fig 4). Bilingual dominance scores were highly correlated with both amount of L1 use (*r* (38) = -.54, *p* <.001, and age of L2 learning *r*(38) = -.55, *p* <.001). Age of L2 learning and amount of L1 use were less correlated *r*(38) = .24, *p* = .064. For word-final schwa duration, amount of L1 use was not a significant predictor, *p* = .079.

#### Nonstructural predictors of spectral qualities

Taken together, our nonstructural predictors significantly predicted the spectral qualities of schwa, defined as the variable F2-F1 *F*(3,39) = 6.23, *p* = .002, *R*^*2*^ = .34. Age of L2 learning was again the single greatest factor for explaining variance in F2-F1 spectral qualities for word-final schwa (*R*^*2*^ = .34, *F* (1,38) = 19.65, *p* <.001). BDS did not quite explain a significant portion of the variance, *F*(1, 39) = 3.78, *p* = .06, *R*^*2*^ = .09. Amount of L1 was again not a significant predictor, *p* = .378. A closer inspection of the spectral qualities reveals that for the dependent variable F1 (height), age of L2 learning also explained the most significant percentage of the variance (*R*^*2*^ = .37, *F*(1, 38) = 23.65, *p* <.001). Bilingual dominance scores also explained a significant portion of the variance for bilinguals’ vowel height (*R*^2^ = .12, *F*(1,38) = 6.03, *p* = .019. As with vowel duration and vowel position (F2-F1), amount of L1 use was highly correlated with age of L2 learning but not a significant predictor on its own.

Likely driven by the heterogeneity observed in F2 formants (anterior-posterior position), all of our predictors had less explanatory power than for duration, F2-F1, and F1 values. Age of L2 learning was the only significant variable for explaining variance along this dimension (*R*^*2*^ = .07, *F* (1,38) = 5.71, *p* = .022). Neither BDS (*p* = .306) nor amount of L1 use (*p* = .037) were significant predictors of native-like vowel qualities. Lack of good fit between our nonstructural predictors and F2 values is consistent with the heterogeneous spread of F2 values by EBs observed in Fig 1.

## Discussion

### Phonetic factors that influence reduced vowel production

Our first research question asked whether EBs and LBs could produce English schwas with native-like durations compared to Miami-based English monolinguals. Given the non-phonemic status of schwa, we anticipated cross-linguistic phonetic interference from L1 Spanish because our bilingual speakers engage in ongoing L1 use. In general, EMs tended not to differentiate between duration of post-labial and post-coronal schwas in word-final position. For our bilingual groups, we discovered that EBs produce post-coronal and post-dorsal schwa with durations that are nearly native-like compared to our EM group. Only post-labial schwa was produced significantly longer by EBs (*M* = .015 seconds). LBs produced longer schwas after consonants in all three places of articulation. However, LBs’ durational hierarchy was the same as EBs where post-coronal schwa < post-labial schwa < post-dorsal schwa.

That EBs very nearly replicated English monolingual norms in certain environments supports the findings of Trofimovich and Baker [107] that experienced L2 learners can produce unstressed syllables with stressed-unstressed duration ratios similar to EMs. On the other hand, a recent study examining Miami’s Cuban Spanish-English bilingual population confirmed that Spanish-English bilinguals clearly differentiate vowel duration between stressed-unstressed syllables when speaking Spanish [108]. This finding appeared to be a stable feature of Miami-Cuban Spanish as it was observed across three generations. The authors of this study suggested that the same stressed-unstressed vowel duration pattern exists in Miami Cuban Spanish as in English, only with less drastic ratios of stressed:unstressed vowel durations. Conversely, it could be true that word-final schwas are truncated in Miami-based EMs as part of a converging “Miami dialect” of English. Further study is needed to determine if the movement is unidirectional (EBs → EMs) or bidirectional (EB↔EMs).

For EMs, the durational hierarchy consisted of statistically equal post-labial and post-coronal schwa durations followed by longer post-dorsal durations. For EBs and LBs, on the other hand, the durational hierarchy consisted of post-labial schwas as the shortest followed by post-coronal schwas, and for both groups post-dorsal schwas were the longest. Post-labial schwas may have been shorter for both bilingual groups due to reduced aspiration accompanying bilinguals’ labial voiceless stops. Since labial voiceless stops are already shorter due to the simplicity of the speech gestures [56], reduced aspiration could further reduce the overall duration of the target syllable containing schwa. Because there are few English words (or truly nativized loanwords) that fit the requirements of our sentence-length stimuli, we had too few tokens available to appropriately analyze this possibility. Future research should anticipate the role of aspiration in elongating or truncating the following vowel.

Vowel qualities and vowel duration are inherently related features, as duration shortens when the vowel is pulled in from the periphery of the oral cavity. As Fig 1 illustrated, LBs tended to produce target schwas with lower F1s and F2s that early bilinguals and monolinguals, although there were four LBs who produced schwa with unusually low F1 and F2 values. Colloquially, even LBs perceive that schwa is different from other vowels in American English, as evidenced when they imitate words like “hamburger.” It may be that the faithfulness observed in most LBs with regard to F1 and F2 formants stems from their mental representation of one reduced vowel as uniform for all unstressed syllables in American English. It was anticipated that this vowel would be located very close to, or at, the low central /a/ vowel of Spanish. It appears to be the case that some LB productions did approximate the /a/ vowel space; however, others were much higher and/or farther back than one would predict of any central vowel. As our nonstructural predictors turned out to be highly correlated, further exploration of the individual differences underlying differentiation of English [ə] and Spanish [a] are needed.

In contrast to the relative faithfulness of LBs’ spectral qualities, EB productions reflected wide variability in their F2 formants. To summarize, some EBs produced anterior F2 formants approximating the space of [e] or the allophone [ɛ], while others produced posterior F2 formants along the same F2 plane as [ɔ]. Given that most words containing word-final schwa end with the letter “a,” this diffusion of F2 formants was unpredicted. Targetlessness for F2 has been proposed in prior monolingual studies, however ([16, 17] but see [8] for contradictory information). If F2 is inherently targetless (i.e. entirely dependent upon co-articulatory effects), it may be the case that EBs are entirely articulating reduced vowels according to monolingual rules. Discrepancies between EMs and EBs under these circumstances would result not from vowel quality mismatches, but from EBs’ failure to produce a feature of the preceding consonant that typically triggers co-articulatory effects during native speaker productions (e.g. aspiration). This hypothesis should be tested using non-word stimuli to explore whether mismatches between EM and EB vowel productions stem from mismatches in the articulation of the preceding consonant and not the target vowel, as prior studies examining these types of questions have heretofore not included bilinguals [8, 12].

### Nonstructural factors and reduced vowel production

In addition to our phonetic features, we examined the role of three nonstructural predictors of native-like reduced vowel production. These three factors were: age of L2 learning, amount of L1 use, and bilingual dominance scores (BDS). For both duration and spectral qualities, we determined that age of L2 learning explained variance in bilingual productions the best. It should be noted, however, that age of L2 learning was a less constrained variable than either bilingual dominance or amount of L1 learning. That is, BDS was constrained by an instrument where all respondents scored from -10 to 10. Amount of L1 use was even more constrained between a score of 0–1, corresponding to the percentage of the day a respondent communicated in Spanish. While amount of L1 use and age of L2 learning were highly correlated, future studies should seek out variables that have more consistent methods of scoring.

The role of bilingual dominance is not often assessed in highly-proficient bilinguals because there is little reason to believe that L2 attainment deficits are causing non-native-like speech. However, our BDS assessment encompassed both qualitative and quantitative measures of reliance on both the L1 and L2, such as emotional attachment and the professional worth attached to a language. Therefore, we determined that a higher personal attachment to English, manifested by higher BDS scores, predicted shorter schwa durations than those produced by speakers who rated Spanish as their preferred language.

Amount of L1 use and age of L2 learning were observed in this study to explain less of the variance for either vowel duration or spectral qualities than age of L2 learning did. However, amount of L1 use has in the past been a significant predictor in studies where subjects are age matched and living in the same country, but differ only along this feature. In a bilingual city such as Miami, it may be that the range of amount of L1 use is too small to explain the variance in our data. For example, no bilingual in this specific environment (who fell within the age range of our study) speaks and/or hears only English or only Spanish.

Other variables worthy of consideration would be “years of education in the L1/L2” and “amount of high-quality input” to be carefully specified. Years of education in the L1/L2 is particularly important because skeptics claim that words such as “sofá” should inevitably be vulnerable to orthographic influence based on reading word-final “a.” To counter, many early and late Spanish-English bilinguals in this environment are educated only in English and/or consider English to be their dominant reading language. Therefore, reading words such as “sofa” may not instinctively activate the Spanish pronunciation when one is used to reading in English. In addition, language instruction from early bilingual school teachers may influence the phonetic norms of our subjects because they operate without English or Spanish monolingual exemplars for most of their lives. As the role of high-quality input is currently under debate [69, 71], it may be that lack of access to monolingual exemplars is a primary contributor to an emerging “Miami dialect” of English that contains Spanish-influenced features [109].

Through the current study we aimed to contribute phonetic data that sociolinguists may find useful for codifying the phonetic properties of an emerging Miami dialect. We discovered that EBs easily master monolingual English vowel reduction patterns but have trouble with pinpointing spectral properties. In contrast, LBs produced a lower, more posterior vowel in word-final position that either EMs or EBs. That both groups of speakers are continuously interacting with a growing group of English monolingual newcomers suggests this interaction will influence the English sound system targeted by future generations of L2-acquiring immigrants who will call Miami home.

## Supporting information

S1 Table Raw durations and spectral values of schwa for all target words produced by each participant. Schwa durations are recorded in seconds and spectral qualities are measured in hertz.
(XLSX)
